# Impact of Spleen Size on Outcomes in Laparoscopic Splenectomy in Children

**DOI:** 10.1155/2015/603915

**Published:** 2015-09-28

**Authors:** Cetin Ali Karadag, Basak Erginel, Ozgur Kuzdan, Nihat Sever, Melih Akın, Abdullah Yıldız, Ali İhsan Dokucu

**Affiliations:** Department of Pediatric Surgery, Sisli Etfal Training and Research Hospital, 34360 Istanbul, Turkey

## Abstract

*Background*. The aim of our study is to compare the efficacy of laparoscopic splenectomy (LS) between enlarged spleens and normal sized spleens. *Methods*. From June 2006 to September 2012, 50 patients underwent LS. The patients consisted of 24 girls and 26 boys with the mean age of 8.64 years (1–18). The patients are divided into two groups according to spleen's longitudinal length on the ultrasonography. Group I consisted of the normal sized spleens; Group II consisted of spleens that are exceeding the upper limit. Groups are compared in terms of number of ports, operative time, rate of conversion to open procedure, and length of hospital stay. *Results*. The mean number of ports was 3.27 and 3.46, the mean length of the operation was 116.36 min and 132.17 min, rate of conversion to open procedure was 9.09% and 10.25%, and the mean length of hospital stay was 3.36 days and 3.23 days, respectively, in Group I and Group II. Although there is an increase in the number of the ports, the operative time, rate of conversion to open procedure, and the length of hospital stay, the difference was not significant between groups (*P* > 0.05). *Conclusion*. LS is safe and effective in enlarged spleens as well as normal sized spleens.

## 1. Introduction

Laparoscopic splenectomy was first performed by Delaitre and Maignien in 1991 [1]. Afterwards, it gained acceptance and became preferred to the traditional open procedure because of the less postoperative pain, shorter hospital stays, quicker wound healing, and better cosmetic results [2]. However, in cases of large spleens, its proper place is still being discussed [3]. The aim of our study is to evaluate the outcome of laparoscopic splenectomy in cases of large spleens.

## 2. Materials and Methods

### 2.1. Patient Selection and Study Design

This is a retrospective cohort study to compare laparoscopic splenectomy in children with normal sized spleens and patients with splenic lengths exceeding the upper limits based on the patients' ages (Table 1). From June of 2006 to September of 2012, 50 patients underwent laparoscopic splenectomy (LS) at our institution. All of them underwent total splenectomy. The demographic findings of the patients are listed in Table 2. Group I consisted of 11 patients with splenic lengths of normal size, and Group II consisted of 39 patients with splenic lengths exceeding the upper limits based on the patients' ages. All patients received pneumococcal, meningococcal, and Haemophilus influenza vaccines preoperatively, and upon discharge, they were given long-acting intramuscular penicillin every 3 weeks for 2-3 years postoperatively.

### 2.2. Technique

Laparoscopic splenectomies (LS) were undertaken in a semilateral position. Under general anaesthesia, a nasogastric tube and a Foley catheter are applied. The patient is placed on his right side in a 15° reverse Trendelenburg position. A 15 mm trocar is inserted through the umbilicus for telescope and the removal of the spleen. A 5 mm port is inserted in the middle point of the line between the umbilicus and xiphoid process for a grasper. A further 5 mm port is placed to the margin of the left pararectal line below the umbilicus. This port is usually used for Ligasure. The intra-abdominal pressure is adjusted to 10–12 mmHg. In 20 patients, one additional port was inserted craniolaterally to the third port. At first, the abdomen is inspected for additional pathologies and for the presence of accessory spleens. The dissection begins with the splenocolic ligament. The adhesions, short gastric vessels, and hilum are divided by using Ligasure, a vessel sealing device (Valleylab, Boulder, CO, USA). All the dissections were performed, without using any other staplers, clips, or coagulation devices. The dissected spleen is placed on the retrieval bag and removed from the umbilical trocar site after fragmentation with ovary forceps. After splenectomy, all patients continued taking antibiotics containing ampicillin-gentamycin for at least three doses. In five patients out of 13, hereditary spherocytosis-concomitant cholecystectomy was also performed because preoperative ultrasonography (USG) revealed gallstones. In those cases, an additional 5 mm port was inserted in the right mid-axillary line to retract the liver; first the splenectomy and then the cholecystectomy are performed in supine reverse Trendelenburg position.

The patients were divided into two groups according to their splenic length based on preoperative ultrasonographic measurements, as described by Rosenberg (Table 1). The first group contained patients with normal splenic sizes, and the second group contained those with splenic lengths beyond the upper limit. Group I and Group II are compared in terms of number of ports, operative time, rate of conversion to an open procedure, and length of hospital stay.

### 2.3. Statistical Analysis

Descriptive statistical methods are used to interpret the groups' characteristics. Kolmogorov-Smirov and Shapiro-Wilk normality tests are used for the normality analysis. ANOVA, Chi-square, and *t*-test methods are used to compare the groups wherever a normal distribution is found. A Kruskal-Wallis test is used for the variance analysis; a Fisher's Exact Test and a Mann-Whitney *U* test are used to compare groups when a normal distribution does not exist. Ninety-five percent is accepted as the confidence interval, and a *P* value ≤ 0.05 is considered to be significant.

## 3. Results

A total of 50 patients underwent laparoscopic splenectomy. Concomitant cholecystectomy for cholelithiasis was performed in five patients (10.2%). In one patient, liver biopsy was performed, and in another patient, a concomitant adrenal mass was excised. Seven accessory spleens were identified and removed in four cases (8%). Five of the laparoscopic splenectomies required conversion to an open procedure because of major bleeding in two patients, dissection problems in two patients, and hypercarbia in one patient (PCO_2_ > 60 mmHg). The mean duration of surgery was 133.4 minutes (range 30–150 min). The comparison of patients in Group I and Group II in terms the demographic findings, the number of ports, operation time, rate of conversion to an open procedure, and length of hospital stay is shown in Table 2.

No mortality or operative morbidity, such as bowel perforation or pancreatic lesion, has happened. No operative bleeding due to Ligasure was recognised, except in the two cases that were converted to open procedures. Two patients required postoperative blood transfusions. There were no wound infections. Follow-up periods varied from 5 months to 6 years, and the patients did not experience any long-term complications.

## 4. Discussion

Extensive splenomegaly creates challenges difficulties regarding laparoscopic removal. The laparoscopic manipulation of an enlarged spleen is difficult because of limited working space, retrieval, and potential trauma to neighbouring organs, vessels, and splenic capsule. In cases of massive splenomegaly, because of the technical problems involved, serious complications such as diaphragmatic rupture and colon perforation have been reported [5]. With the development of technology and experience in technical issues, these difficulties have been resolved [6]. In the last decade, laparoscopic splenectomy has become accepted by surgeons as a safe and effective alternative to open surgery, even in children, regardless of the size of the spleen [7, 8]. Thirty-nine of our patients had splenic lengths exceeding the suggested upper limit of normal according to Rosenberg's study [4]. The number of ports, the operative time, the necessity of conversion to open procedure, and the mean length of hospital stay did not have any correlation with the size of the spleen in our series.

The dissection of the splenic hilum is the most important step in LS. We performed all our dissections with Ligasure which may reduce the operating time and blood loss, as was confirmed by our results [8, 9]. Gelmini et al. presented 63 LSs in which they performed the entire dissection with Ligasure and reported very low levels of bleeding. They preferred this device because of the safety and time saved [10]. Misawa et al. reported 87 LSs in which they dissected all splenic ligaments and vessels with Ligasure, which reduced intraoperative blood loss [11].

HS (hereditary spherocytosis) and other hematological disorders are the main indications of LS [12]. In our series, the rate of thalassemia was relatively higher due to its high prevalence in our country.

All operations are performed with the patient remaining supine in a 45° right lateral reverse Trendelenburg position, as Mattioli et al. described [13]. Positioning and stabilization of the patient are facilitated by the use of a beanbag mattress and various rolls and pads. The patient is positioned with the umbilicus at or near the break in the table. This allows more distance between the lower ribs and iliac crest when the table is flexed. We believe that a semilateral position leads to good exposure, especially in the hilar and medial region, where accessory spleens are frequently found. This procedure allows the abdominal organs to be kept away from the splenic hilum, providing a good exposure. First, the splenic artery is ligated, and then the splenic vein is ligated. This avoids the peroperative swelling of the spleen and prevents bleeding because of distention from the splenic surface. Finally, the lateral and diaphragmatic ligaments of the spleen are divided. The reason for dividing them at the end of the dissection is preventing the spleen from pulling down.

The detection of accessory spleens is crucial because of the risk of recurrence in cases of haematologic disease. The exploration of the abdomen should be performed at the beginning of the operation, as Vecchio et al. described in their series of 18 patients [14]. The accessory spleens can be easily missed during the operation.

The mean operative time is a parameter for the success of LS. Shorter operative times may provide fewer lung infections, less pain, fewer complications, and shorter hospital stays. Rescorla and Duffy have reported 200 LSs with a mean operative time of 115 minutes, whereas the mean operative time of the last 50 cases in this series was 86 minutes [15]. The mean operative time for our first 25 cases was 154.6 min, whereas the mean operative time for our last 25 cases was 115.5 minutes. Increasing experience with laparoscopy allowed us to speed up the surgery. Furthermore, Ligasure permitted us to engage in more convenient surgery.

Mattioli et al. compared LS and open splenectomies in terms of quality of life and cosmetics [16]. They favoured the laparoscopic approach because of its good cosmetic results and the reduction in hospital stay, abdominal wall traumatism, and postoperative pain. We suggest laparoscopic splenectomy should be used because of its better cosmesis, shorter hospital stays, and lesser postoperative pain.

The limitations of our study are the limited number of patients and being at the beginning of the learning curve of the surgeons. The single center design of study avoids the impact of different surgents with differing degrees of experince.

## 5. Conclusion

LS is preferred because of its less postoperative pain, good cosmesis, and shorter hospital stays. Ligasure can be sufficient for all of the dissections of LS. The size of the spleen does not have an impact on the surgical approach, either laparoscopic or open splenectomy.

## Figures and Tables

**Table 1:** Age and suggested upper limit of the length of the spleen, according to Rosenberg's study [4].

Age	Suggested upper limit
0–3 months	6 cm
3–6 months	6.5 cm
6–12 months	7 cm
1-2 years	9 cm
2–4 years	9 cm
4–6 years	9.5 cm
6–8 years	10 cm
8–10 years	11 cm
10–12 years	11.5 cm
12–15 years	12 cm
Female 15–20 years	12 cm
Male 15–20 years	13 cm

**Table 2:** The comparison of patients in Group I (patients with normal sized spleens) and Group II (patients with splenic lengths exceeding the upper limits based on the patients' ages) in terms of patients' demographic data, the number of ports, operation time, rate of conversion to an open procedure, and length of hospital stay.

	Group I (*n* = 11)	Group II (*n* = 39)	*P*
Gender			
Female	5	19	
Male	6	20	
Age (y)	9.72	6.12	
Diagnosis			
Hematologic			
HS	3	15	
ITP	7	6	
*β*-thal.		13	
AHA	1	1	
Splenic cysts		2	
Unknown origin		1	
Lymphangioma		1	
Mean number of ports	3.27	3.46	0.26
Mean length of the operation (min)	116.36	132.17	0.31
Rate of conversion to open procedure	1/11	4/39	0.70
Mean length of hospital stay (days)	3.36	3.23	0.81

The difference between the number of the ports, the operative time, rate of conversion to an open procedure, and length of hospital stay was not significant for Group I and Group II (*P* ≤ 0.05) (HS: hereditary spherocytosis, ITP: idiopathic thrombocytopenic purpura, *β*-thal.: *β*-thalassemia, and AHA: autoimmune hemolytic anemia).
